# Adrenal Ganglioneuroma Presenting As Left Renal Mass

**DOI:** 10.14740/wjon783w

**Published:** 2014-05-06

**Authors:** Hakan Ozturk

**Affiliations:** a Department of Urology, Sifa University School of Medicine, Izmir, Turkey

**Keywords:** Ganglioneuroma, Adrenal ganglioneuroma, Incidentaloma, Immunohistochemistry, Renal mass

## Abstract

Ganglioneuromas (GNs) are benign tumors resulting from neural crest tissue. GNs contain mature ganglion cells and Schwann cells. GNs most commonly occur in the retroperitoneum and posterior mediastinum. GNs rarely occur in the adrenal gland. A 45-year-old asymptomatic patient presented with an incidental finding of left renal mass. A 10 cm mass lesion located in the upper pole of the left kidney and lymphadenopathy in renal hilus were detected. The patient underwent transperitoneal radical nephrectomy involving the removal of left adrenal gland. The immunohistochemical examination showed strong positive staining for S100, neuron-specific enolase, synaptophysin and chromogranin. The diagnosis of mature GN was established. GNs are among the rare diseases that should be considered in the evaluation of renal masses, particularly in the differential diagnosis of upper pole tumors of the kidneys. It can be confused with renal cell carcinomas.

## Introduction

Ganglioneuromas (GNs) are rare benign tumors, which may mature from neuroblastomas or may arise *de novo*. Adrenal GNs arise from the neuroblasts in the adrenal medulla. GNs account for 0-6% of the tumors occurring in the adrenal medulla [1]. These tumors are rare neurogenic tumors that derived from sympathetic ganglion cells, peripheral sympathetic nerves and less commonly from the neuroblasts in the adrenal medulla. GNs are slow-growing tumors mostly detected as an incidental finding, and the patients are usually asymptomatic. The symptoms occur in patients where the tumor growth has reached a significant degree and caused compression on the neighboring tissue. The tumor can also cause serious symptoms when they expand to a significant extent. These tumors most commonly occur in the posterior mediastinum and retroperitoneal area. Treatment involves the surgical removal of the tumor mass [2, 3].

## Case Report

### Clinical features

A 45-year-old female patient presented with a left renal mass detected as an incidental finding. The patient was asymptomatic. The results of biochemical tests were as follows: glucose 95 mg/dL, creatinine 0.9 mg/dL, urea 28 mg/dL; AST, ALT, ALP and GGT were within normal ranges; WBC 5.41 × 10^3^/µL and Hgb 13.7 g/dL. USG and CT showed a 100 × 71 × 66 mm heterogeneous solid mass located in the upper pole of the left kidney and lymphadenopathy in the renal hilus, and the patient subsequently underwent transabdominal radical nephrectomy including ipsilateral adrenal gland with the prediagnosis of renal cell cancer (RCC). The diagnosis of mature adrenal GN was established, and the tumor was hormonally inactive.

### Immunohistopahtologic features

**Figure 1:**
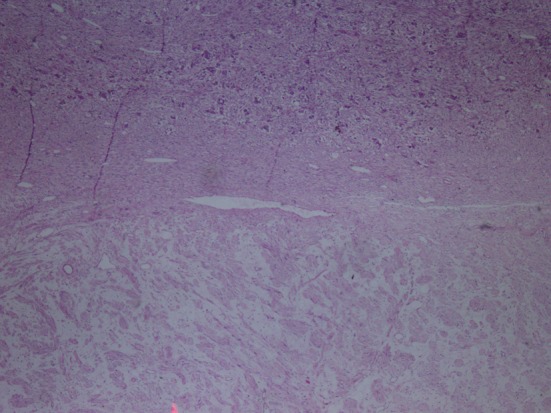
Hematoxylin-eosin staining demostrating H&E (× 40).

**Figure 2:**
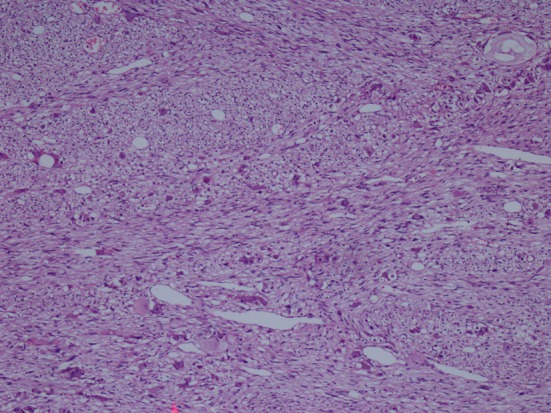
Hematoxylin-eosin staining demostrating H&E (× 100).

**Figure 3:**
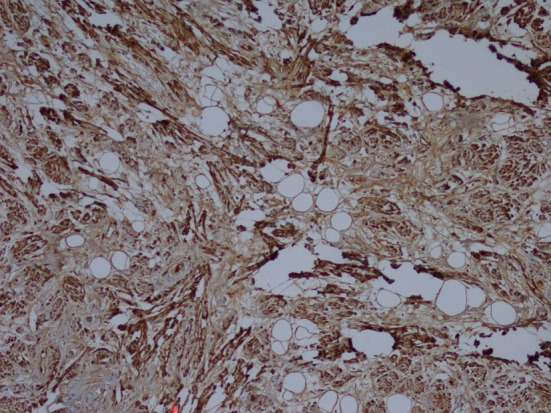
Immunohistochemistry showing S100 (× 100).

Macroscopic examination of the radical nephrectomy material measuring 18 × 7 × 5 cm showed a solid, gray-white colored tumor measuring 10 × 7 × 3 cm, located in the upper pole of the kidney but without any direct relation to the kidney. Focal adrenal tissue was observed in the cross-section of the tumor. The kidney and surgical margins were tumor-free. Three lymph nodes measuring 1 cm in diameter were detected in the renal hilus. The microscopic examination revealed mature ganglion cells in Schwannian stroma. The immunohistochemical examination showed strong positive staining for S100, neuron-specific enolase, synaptophysin and chromogranin. These three lymph nodes are considered to be reactive lymph nodes (Fig. 1-3).

### Treatments and survival

The patient did not develop a secondary focus or local recurrence at 96 months after surgery involving left transperitoneal radical nephrectomy plus adrenalectomy.

## Discussion

GNs are benign tumors that derived from the neural crest, and they may arise “*de novo*” or they mature from a neuroblastoma. The tumor most commonly occurs in the retroperitoneal area (41.5%) and posterior mediastinum (37.5%), but may also occur in the adrenal tissue (21%) [4, 5]. GNs rarely occur in patients younger than 30 years of age. The incidence increases with age [6]. The incidence rate is 0.2% in young individuals and rises up to 3% at the age of 50 and 7% at 70 years [7].

According to the international neuroblastoma pathology classification (the Shimada system), GNs are Schawannian stroma-dominant tumors. The tumor is divided into two subtypes: mature and immature. However, full maturation of the ganglion cells is a rare condition. Of all GNs, 7% is of the mature subtype [8]. The present case had GN of the mature subtype. As a composite tumor, the co-occurrence of adrenal GN and pheochromocytoma has been reported in the literature. A composite tumor may arise in the co-occurrence of neoplastic chromaffin cells along with GN developing under possible influence of neural growth factors. These composite tumors are hormonally active tumors. It is not known which tumor differentiated earlier and triggered the other [9].

Thirty-nine percent of the GNs secrete catecholamines [2]. Elevated catecholamine levels result in hypertension, diarrhea, sweating and flushing. Urinary levels of vanillin mandelic acid and homovanillic acid are found elevated. The vast majority of the patients do not show hormonal activity, as was the case in the present patient. Therefore, quantitative analysis of vanillin mandelic acid/homovanillic acid is not indispensable for the differential diagnosis of GN in this region [5].

The widespread use of imaging methods such as US, CT and MRI has increased the incidence of GN [10]. Adrenal GNs are hormonally inactive silent tumors. They therefore exhibit an asymptomatic course and they can reach large sizes [11]. The radiological appearance of GNs resembles to that of adrenal tumors, adrenocortical carcinoma, pheochromocytoma and RCCs arising in the upper pole of the kidney [12].

GNs have no specific signs or symptoms, and this complicates establishing a diagnosis before pathological examination can take place. CT and MRI are important methods that determine the size, location and composition of the mass and the surgical plan. Identification of the relation with the neighboring tissue helps the selection of an appropriate surgical method. CT may show calcification; however, only 60% of GNs show calcification. MRI is superior to CT in diagnosing GN. However, no single radiological finding has ultimate power to differentiate RCC from an adrenal mass [5]. In the literature, one study reported a misdiagnosis rate of 64.7% for CT and MRI performed before the operation [13]. In the present case, differentiating the lesion from RCC was challenging due to the location of the mass in the upper pole, hormonal inactivity and the presence of lymphadenopathy in renal hilus. It should be recognized that retroperitoneal GNs may occur synchronously in multiple foci. Ishida et al reported numerous hormonally active adrenal GNs that mimic lymphadenopathy in periaortic location [14]. Complete surgical excision is particularly recommended in the treatment. Recurrence is, however, rare even in the case of incomplete excision. Retrosi et al performed incomplete resection and did not report recurrence after 40 months of follow-up. However, close follow-up is recommended in patients with GN [15]. The present case remained disease-free during the follow-up period of 96 months.

### Conclusion

Adrenal GNs are benign tumors that may occur almost in all age groups. There is no specific CT or MRI finding leading to the diagnosis. The tumor can be hormonally active but can also be totally inactive. The diagnosis of GN should be considered in asymptomatic retroperitoneal masses reaching large sizes. The tumor can be confused with other mass lesions of the adrenal glands and also with renal masses. The treatment involves surgical excision. The diagnosis can only be established by immunohistopathological examination.
